# Supplementary material for: SUMOylation of zebrafish transcription factor Zbtb21 affects its transcription activity
Source: PeerJ. 2024 Apr 22;12:e17234. doi: 10.7717/peerj.17234 (PMC11044885; doi:10.7717/peerj.17234)

**FIGURE1：**

**WB-** **HA-Zbtb21：**


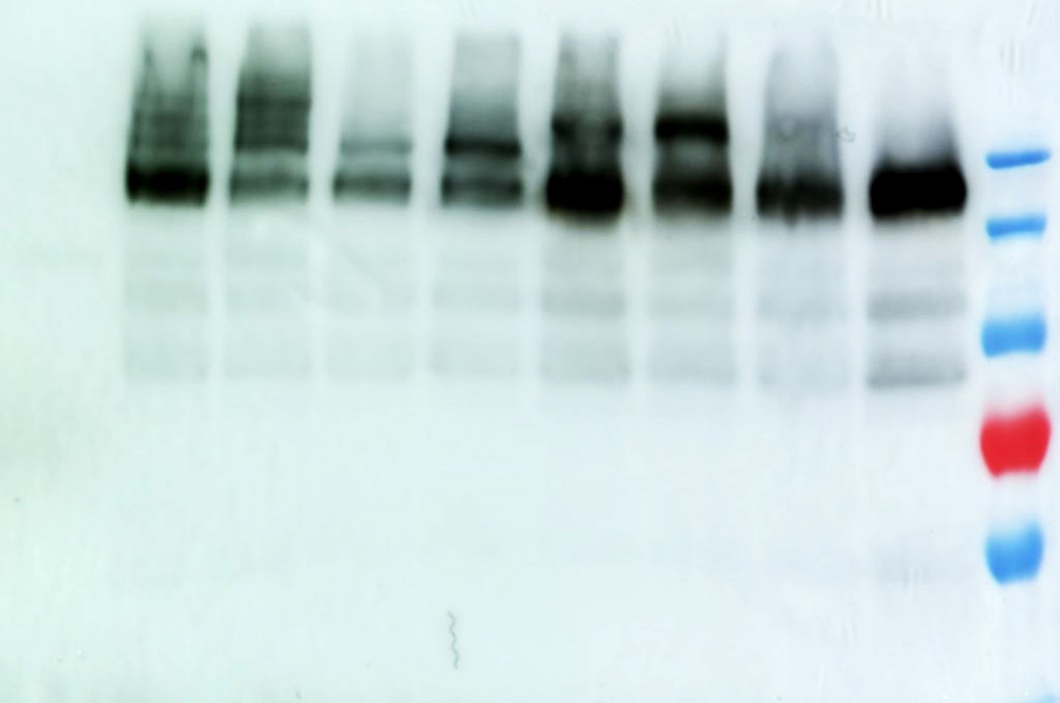


WB-TUBULIN:


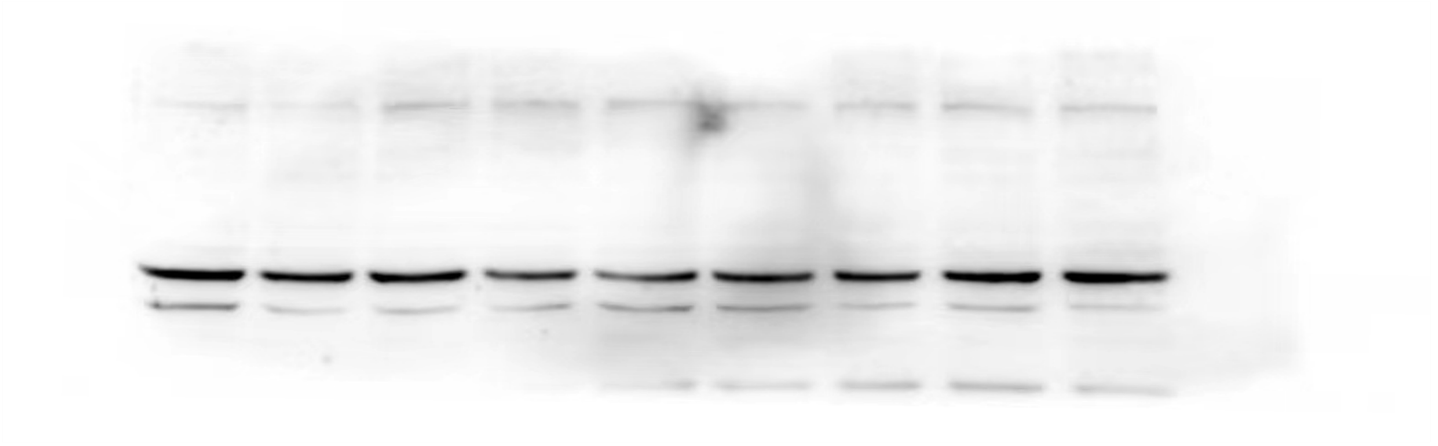


**IP：HA-Zbtb21 - SUMO1**

IP-anti-sumo1：





Input-anti-HA：





**FIGURE2：**

| zbtb14-promoter | PCS2 | | | HA-Zbtb21 | | | HA-Zbtb21^K419R^ | | | HA-Zbtb21^K845R^ | | | HA-Zbtb21^K419+845R^ | | |
| --- | --- | --- | --- | --- | --- | --- | --- | --- | --- | --- | --- | --- | --- | --- | --- |
|  | 1 | 1 | 1 | 1.174548 | 1.165457 | 1.127235 | 1.243509504 | 1.284502 | 1.400117 | 1.296476588 | 1.293151 | 1.372978 | 1.507881317 | 1.323127 | 1.251392 |

| CDC6-promoter | PCS2 | | | | HA-Zbtb21 | | | | HA-Zbtb21^K419R^ | | | | HA-Zbtb21^K845R^ | | | | HA-Zbtb21^K419+845R^ | | | |
| --- | --- | --- | --- | --- | --- | --- | --- | --- | --- | --- | --- | --- | --- | --- | --- | --- | --- | --- | --- | --- |
|  | 1 | 1 | 1 | 1 | 0.669233 | 0.723288 | 0.785301677 | 0.904241 | 0.949006 | 0.824822847 | 0.901663 | 0.749413 | 0.73787838 | 0.789528 | 0.633254 | 1.069653 | 1.059079 | 0.819347 | 0.931963 | 1.073659 |

| pu1-promoter | PCS2 | | | HA-Zbtb21 | | | HA-Zbtb21^K419R^ | | | HA-Zbtb21^K845R^ | | | HA-Zbtb21^K419+845R^ | | |
| --- | --- | --- | --- | --- | --- | --- | --- | --- | --- | --- | --- | --- | --- | --- | --- |
|  | 1 | 1 | 1 | 0.75713628 | 0.701457293 | 0.75543886 | 0.875172652 | 0.935366347 | 1.000300075 | 0.798572744 | 1.11395223 | 1.081470368 | 0.861187845 | 1.097490217 | 1.09587397 |

WESTERN-ANTI-HA


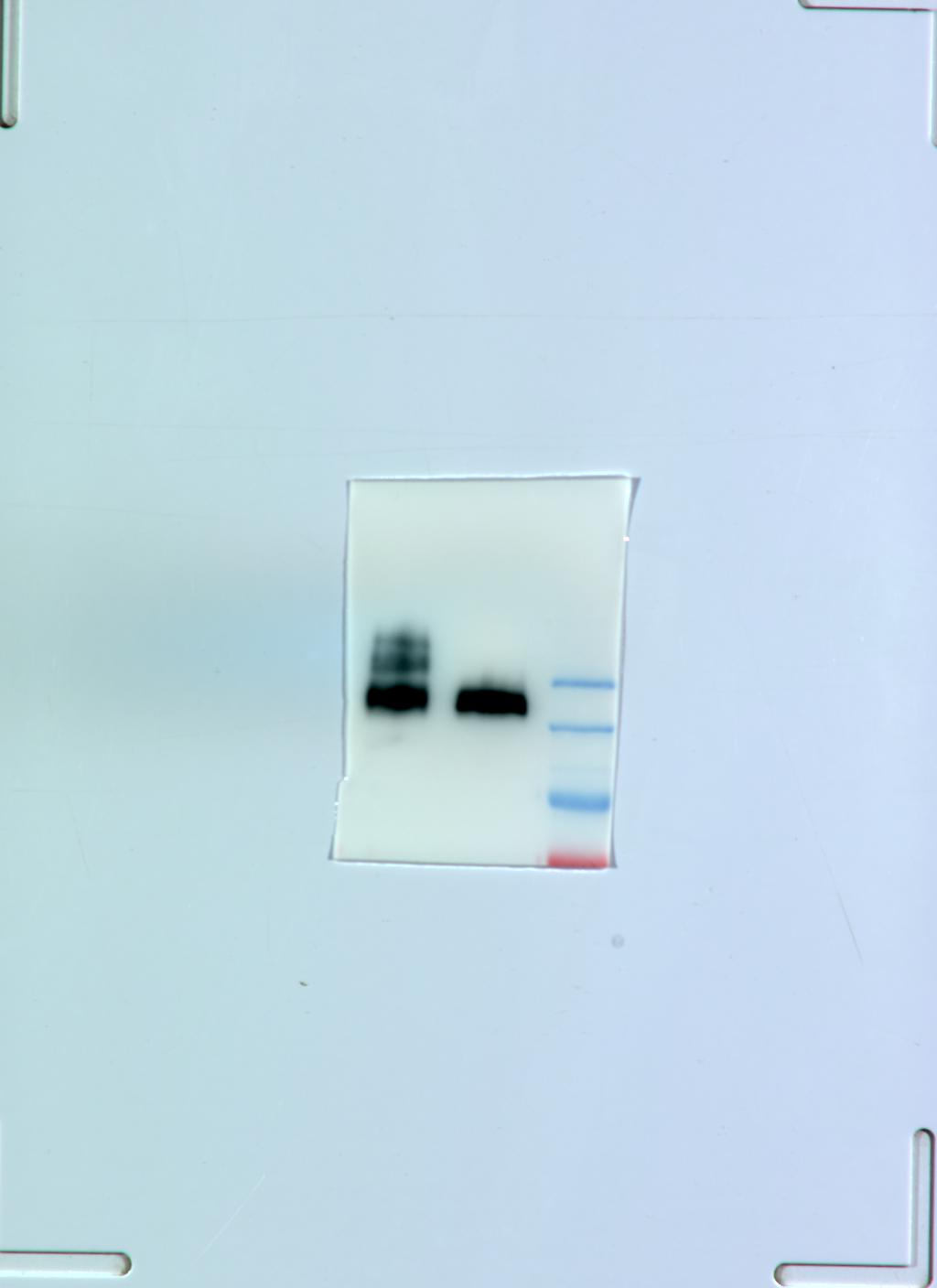


WESTERN-ANTI-TUBULIN


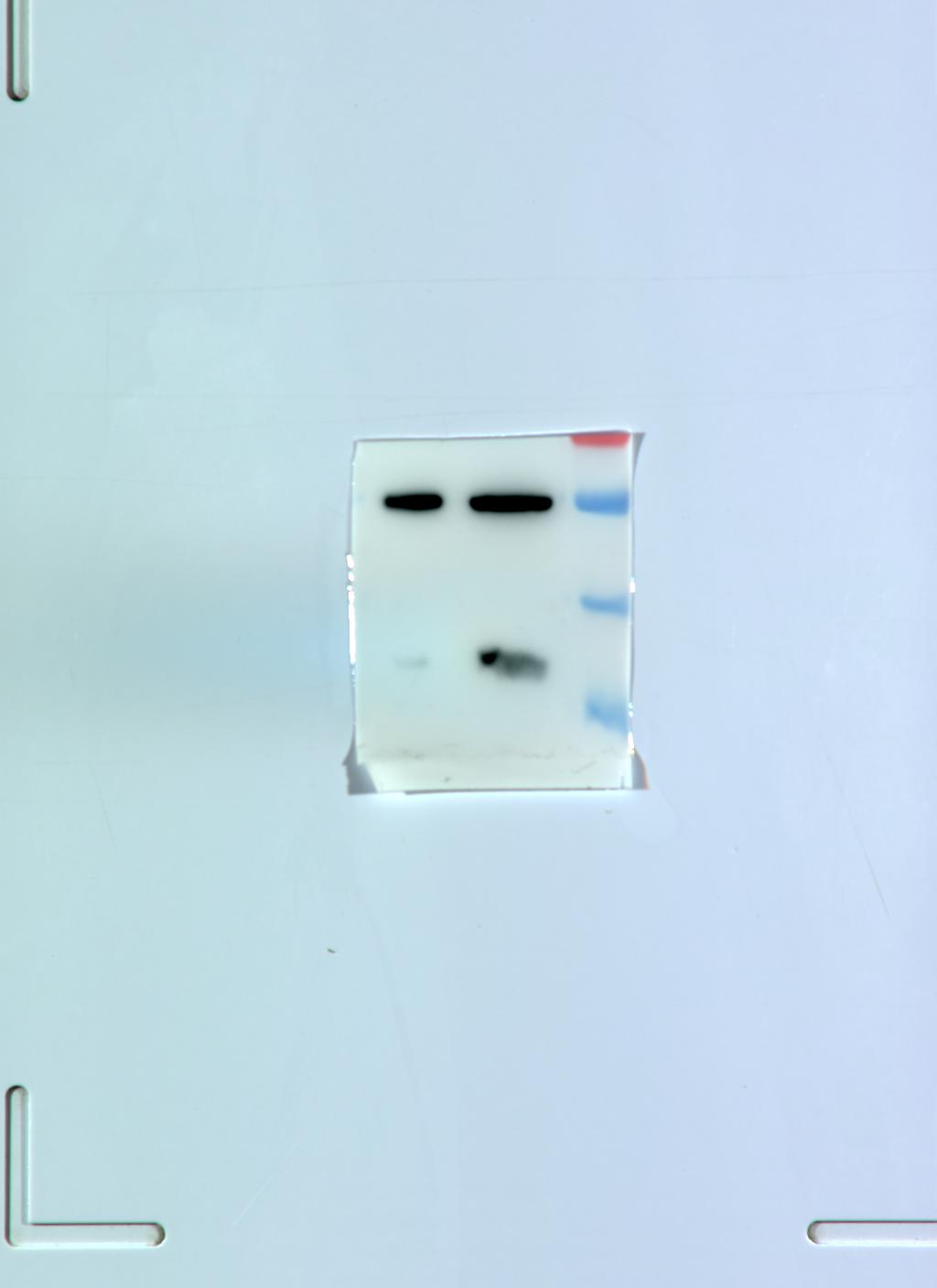


INPUT-ANTI-BRD4


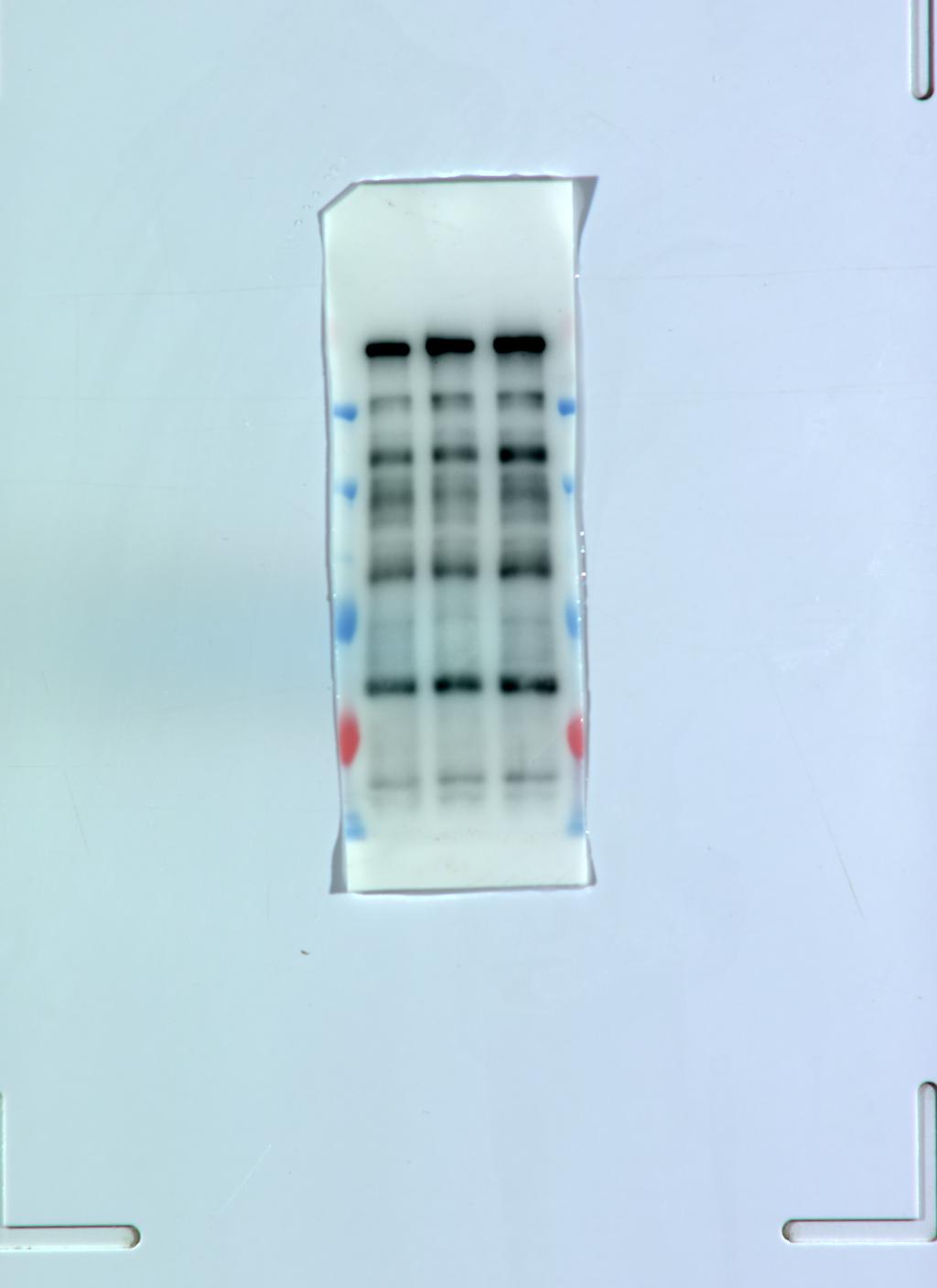


INPUT-ANTI-HDAC4


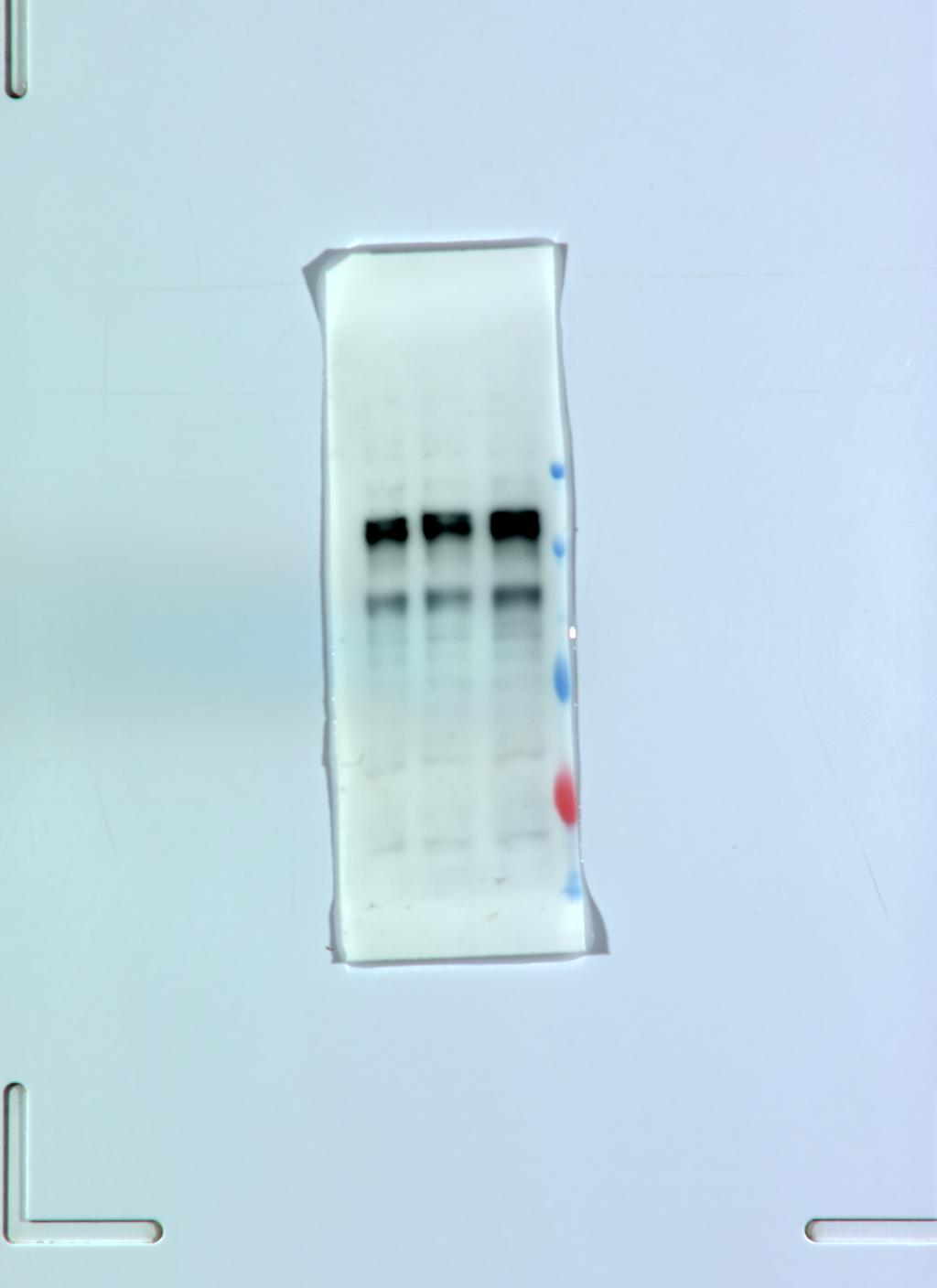


INPUT-ANTI-HA


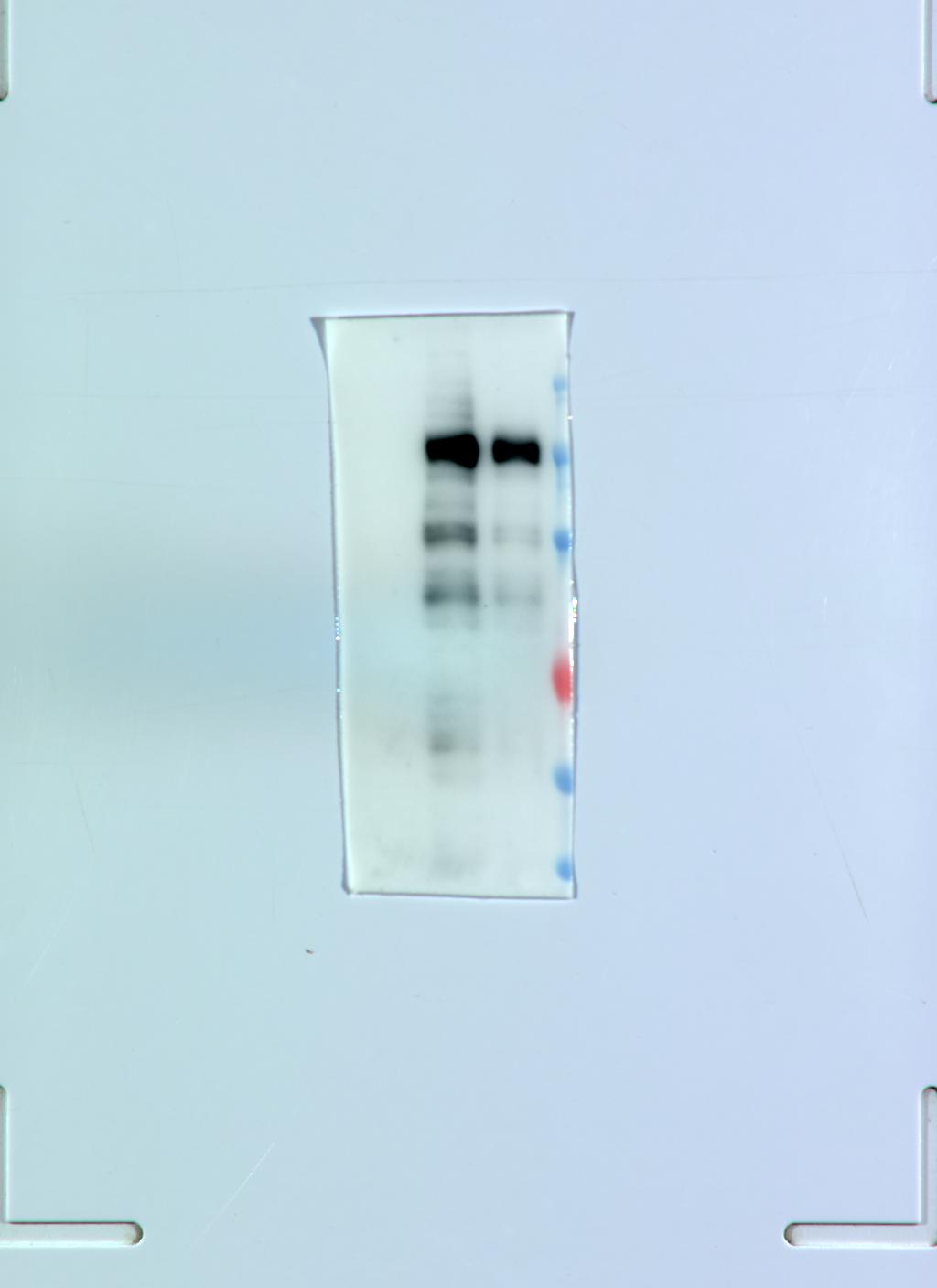


IP-ANTI-BRD4


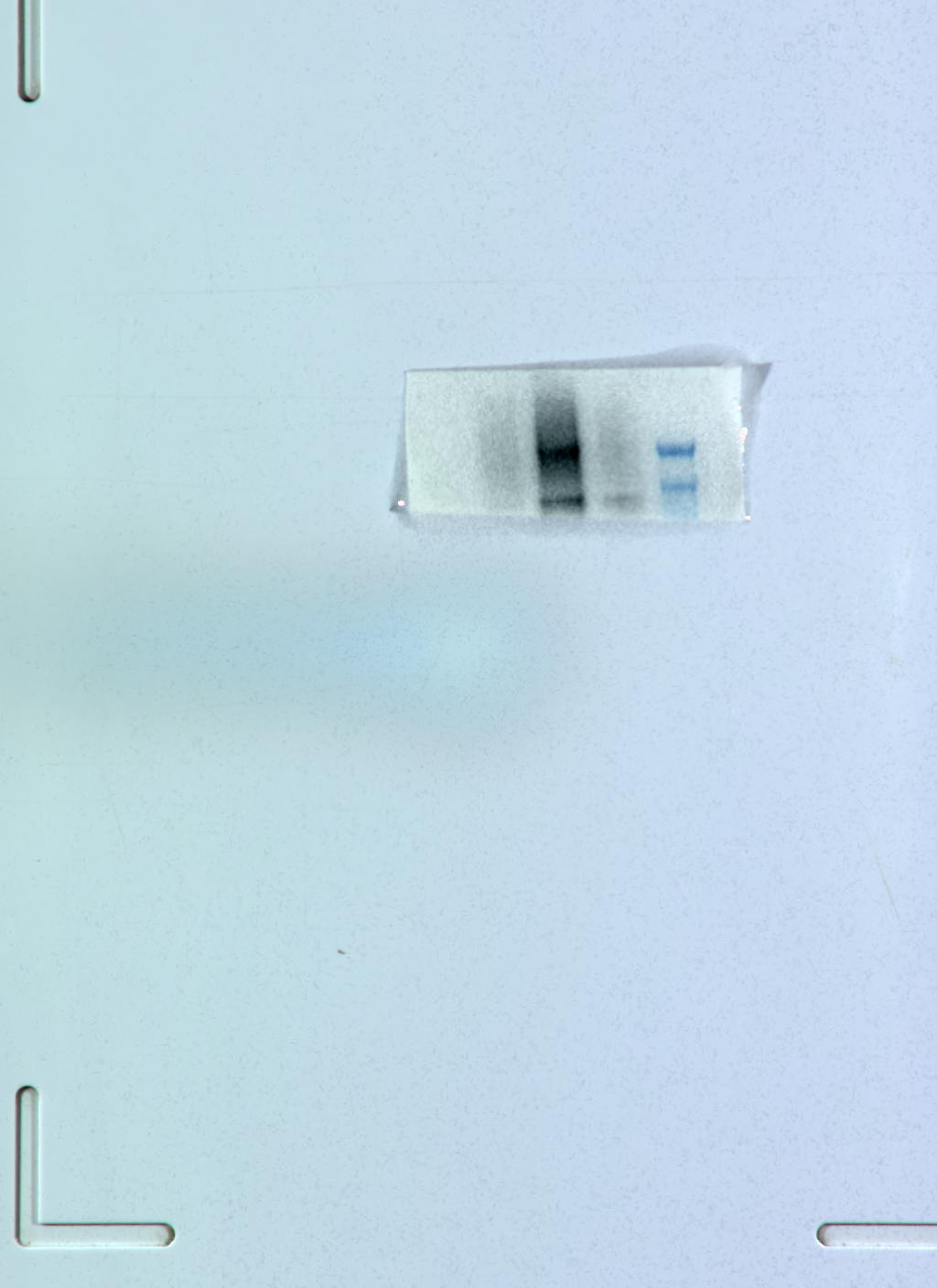


IP-ANTI-HDAC4


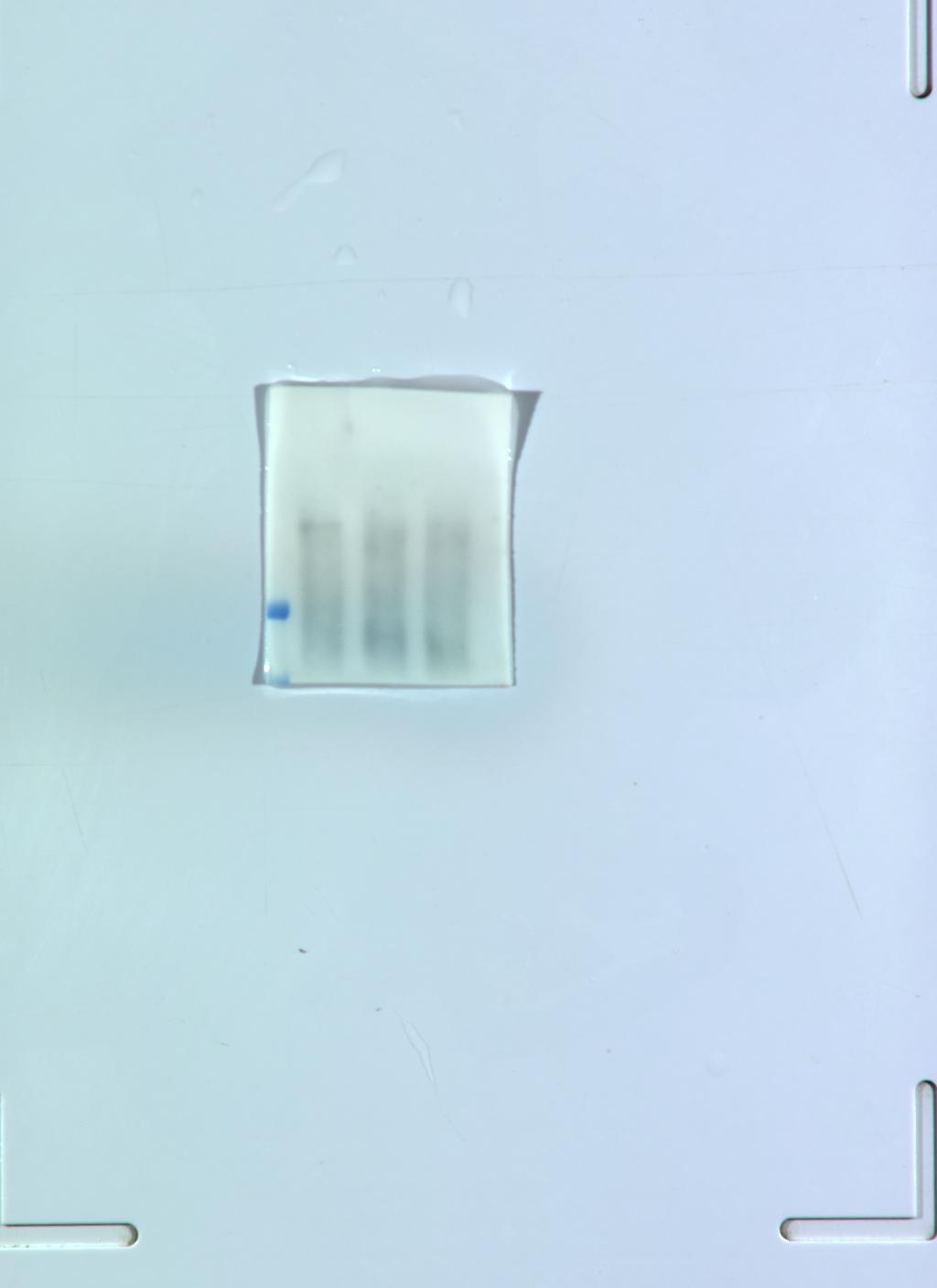


**FIGURE3:**

**PCS2:**

DAPI:


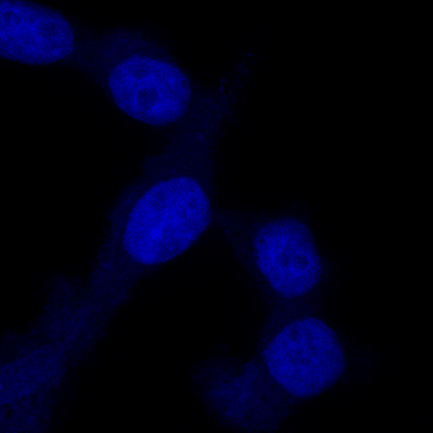


HA:


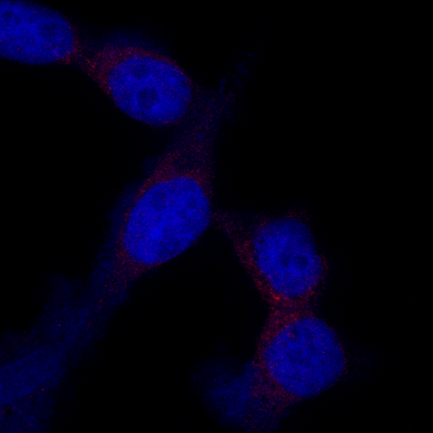


MERGE:


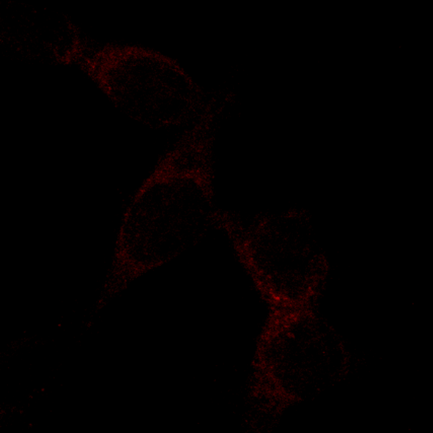


**HA-Zbtb21:**

DAPI:


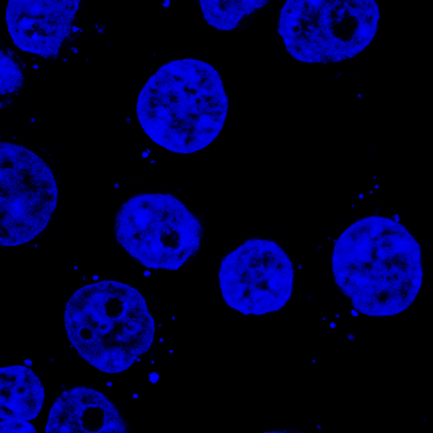


HA:


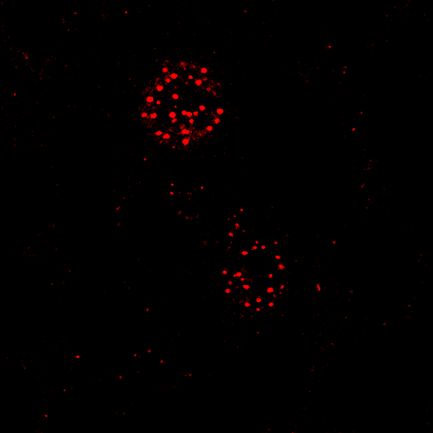


MERGE:


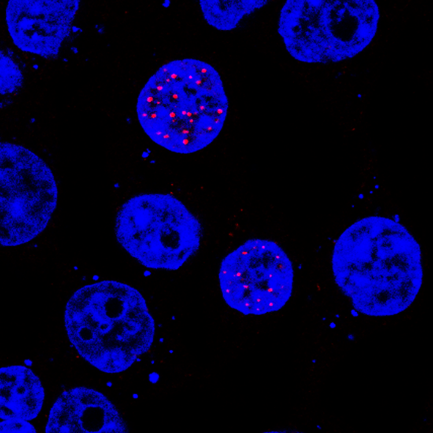


**HA-Zbtb21^K419R^:**

DAPI:


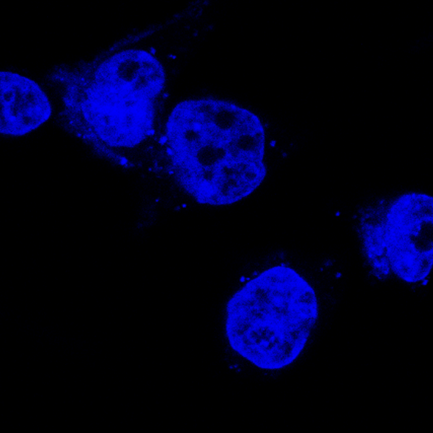


HA


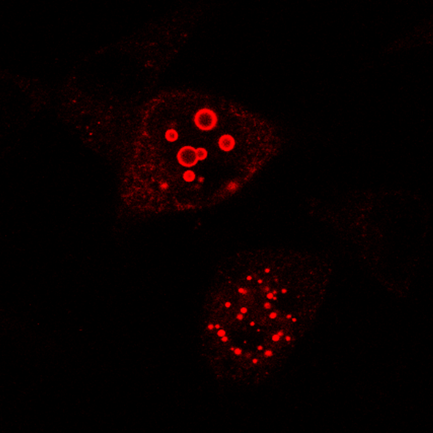


MERGE


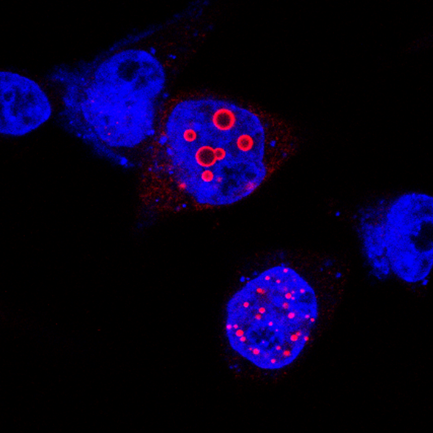


**HA-Zbtb21^K845R^:**

DAPI:


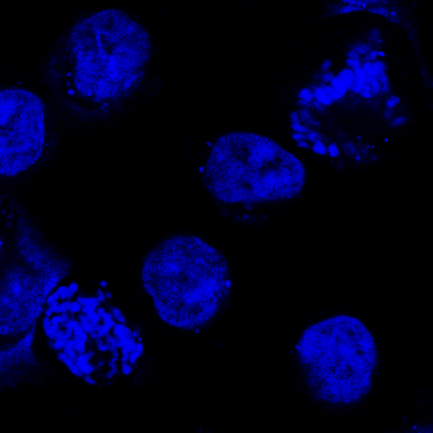


HA:


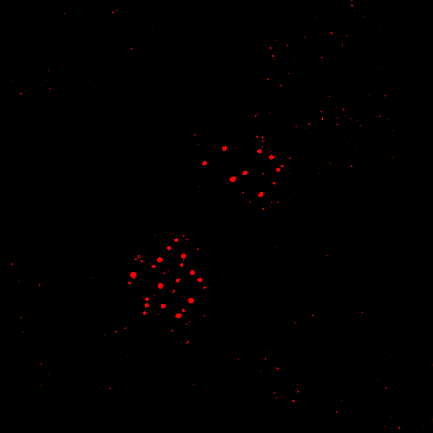


MERGE:


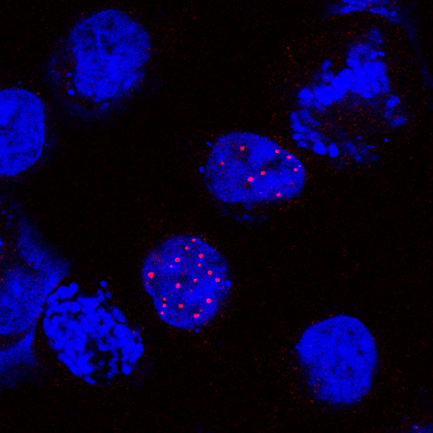


**HA-Zbtb21^K419+845R^:**

DAPI:


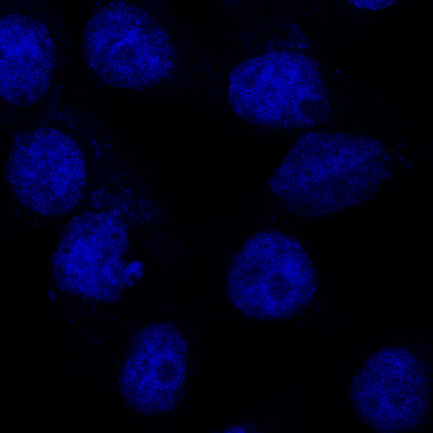


HA:


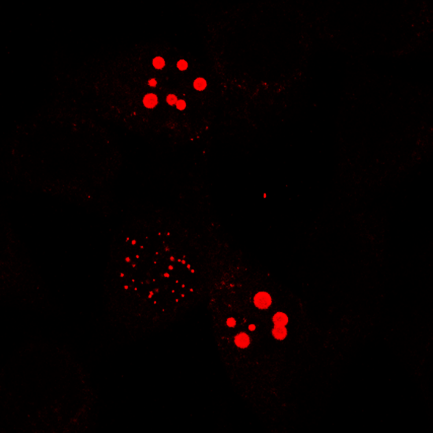


MERGE:


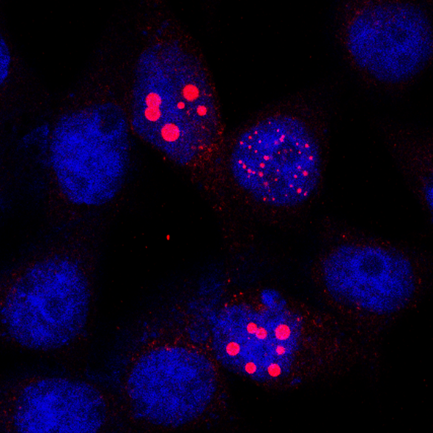


**FIGURE4:**

**IP:** **HA-Zbtb21-Zbtb14**

IP-Anti-HA


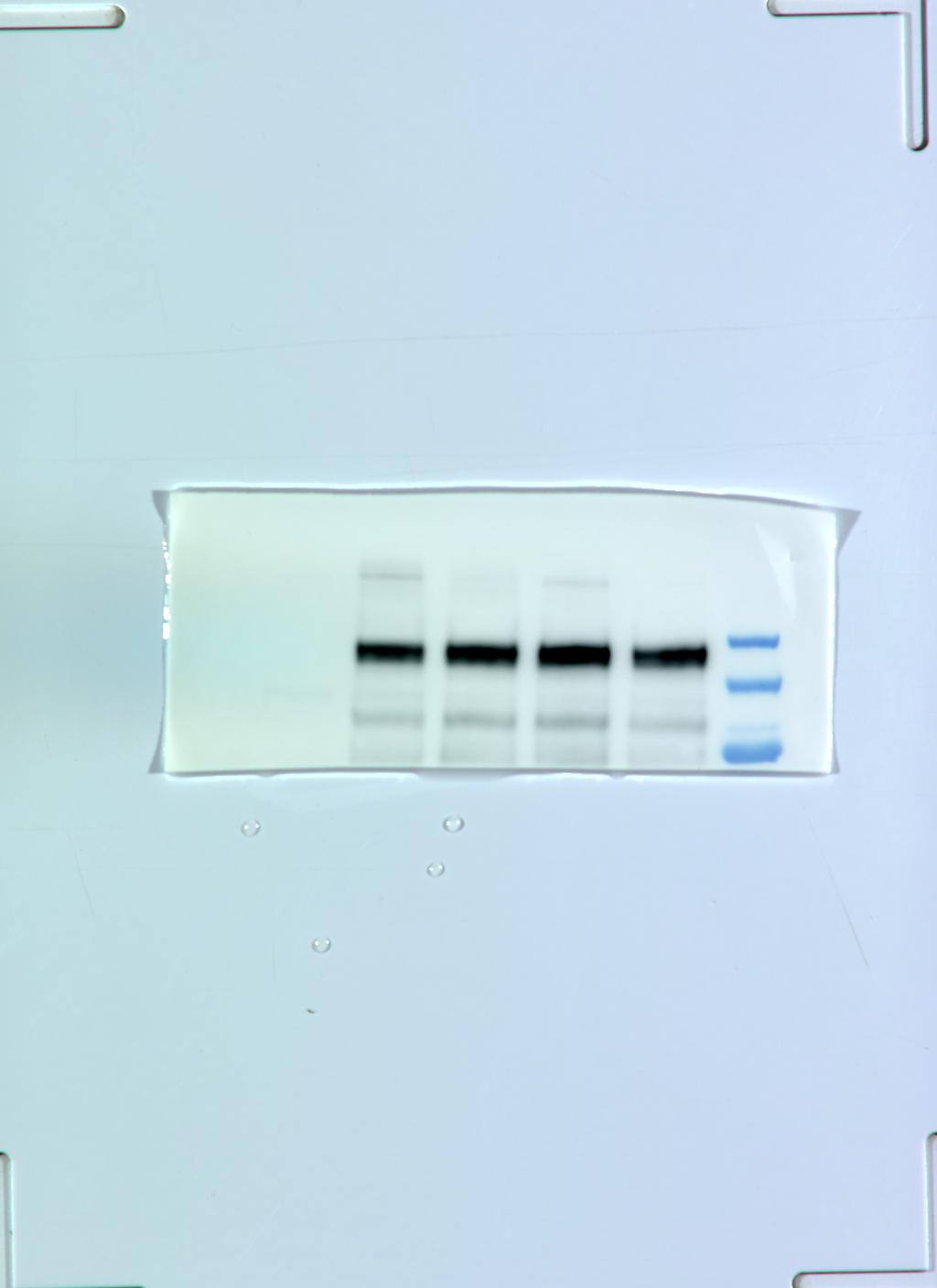


IP-Anti-FLAG


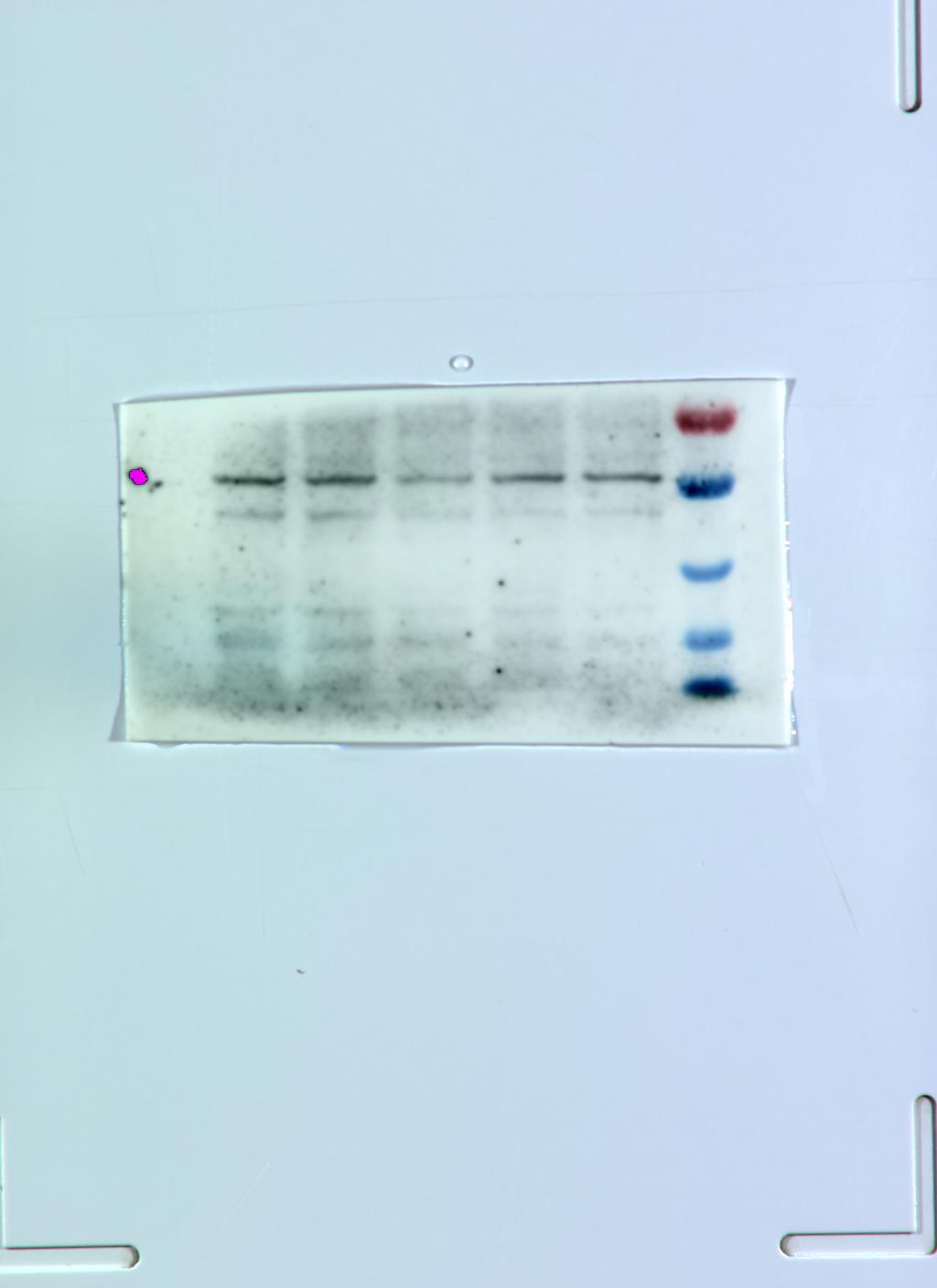


INPUT- anti-HA


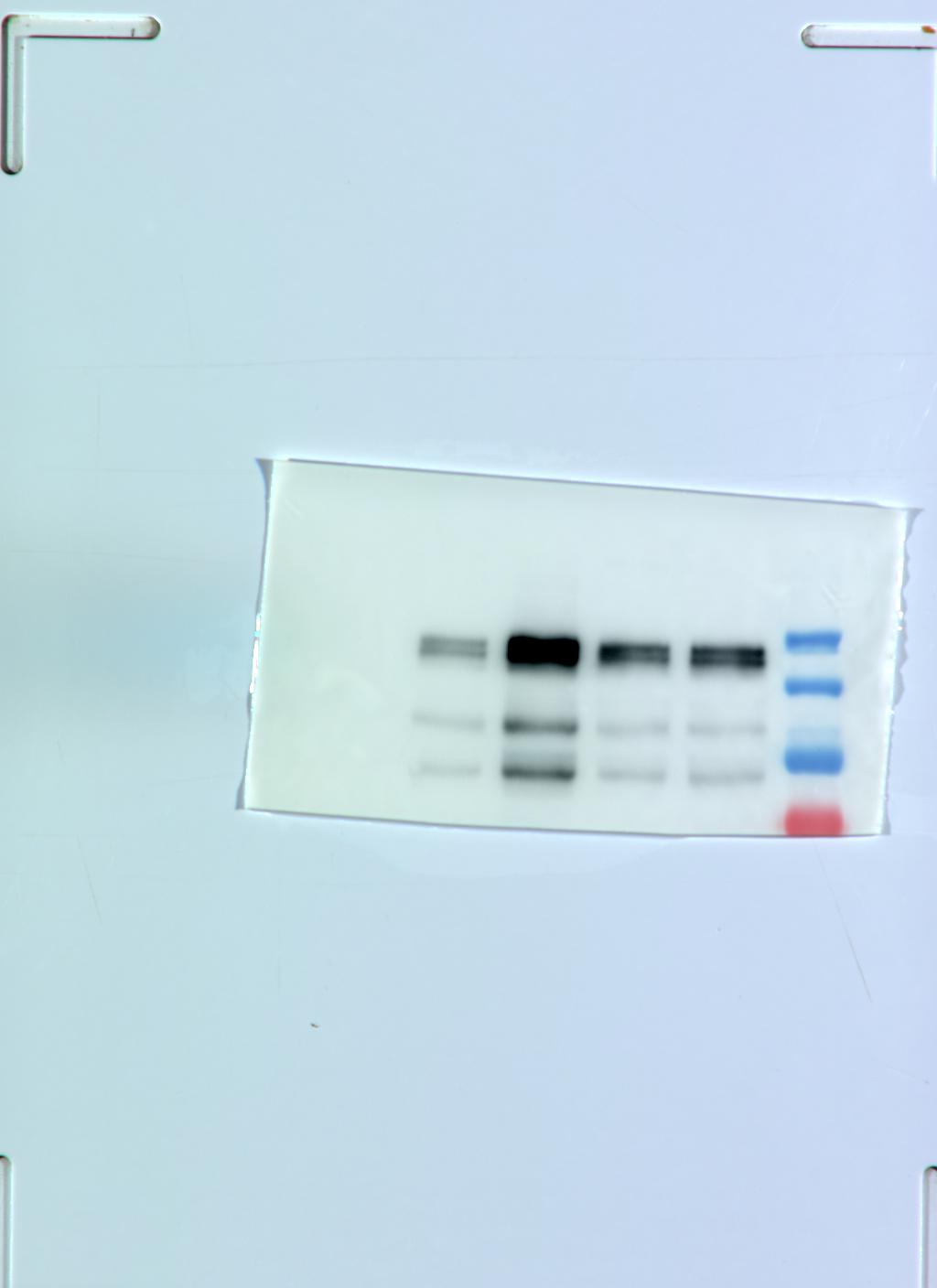


INPUT- anti-flag


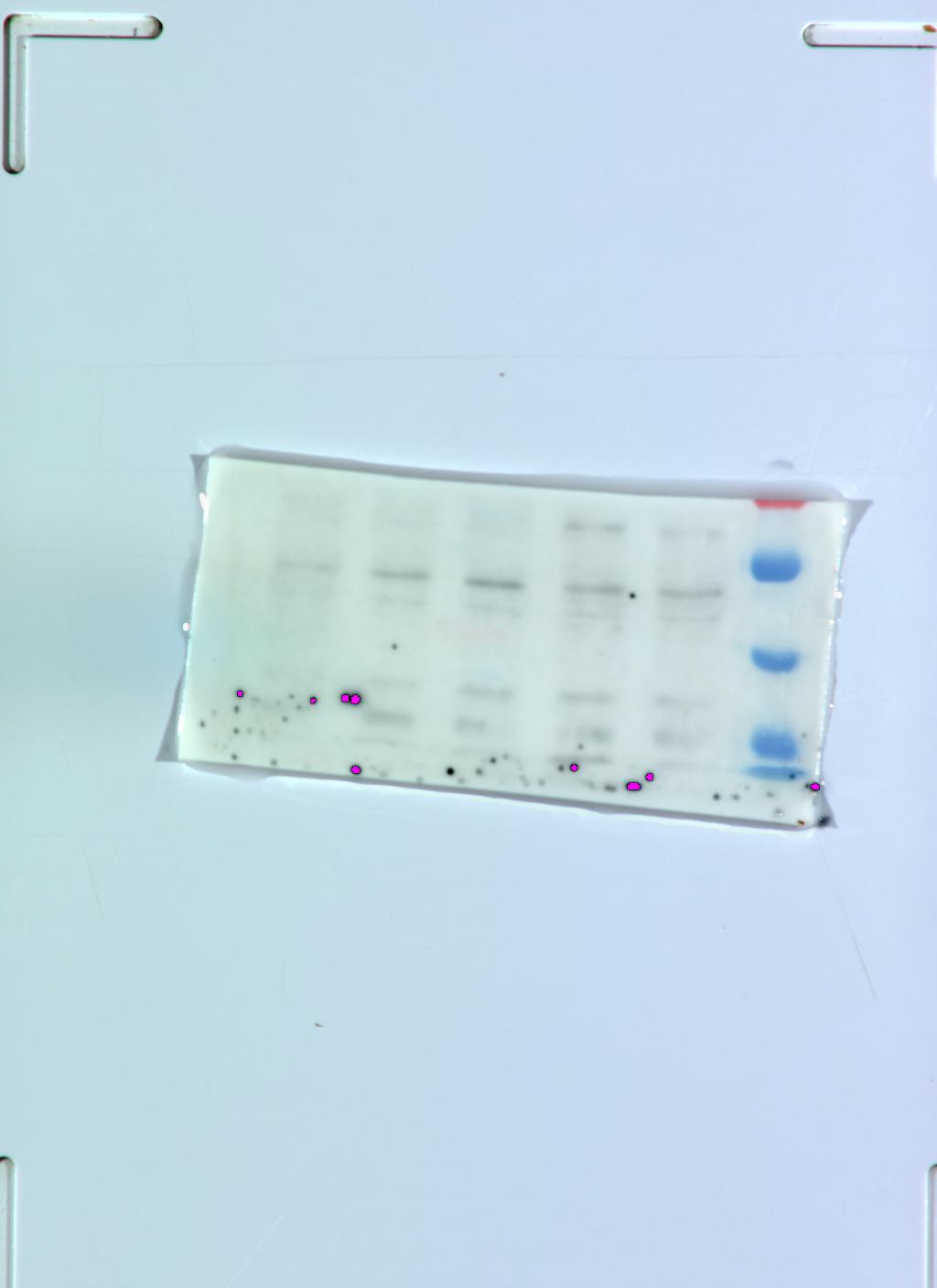


**IP: HA-Zbtb21^k419+845R^-FLAG-Zbtb21^K419+845R^**

IP-anti-HA


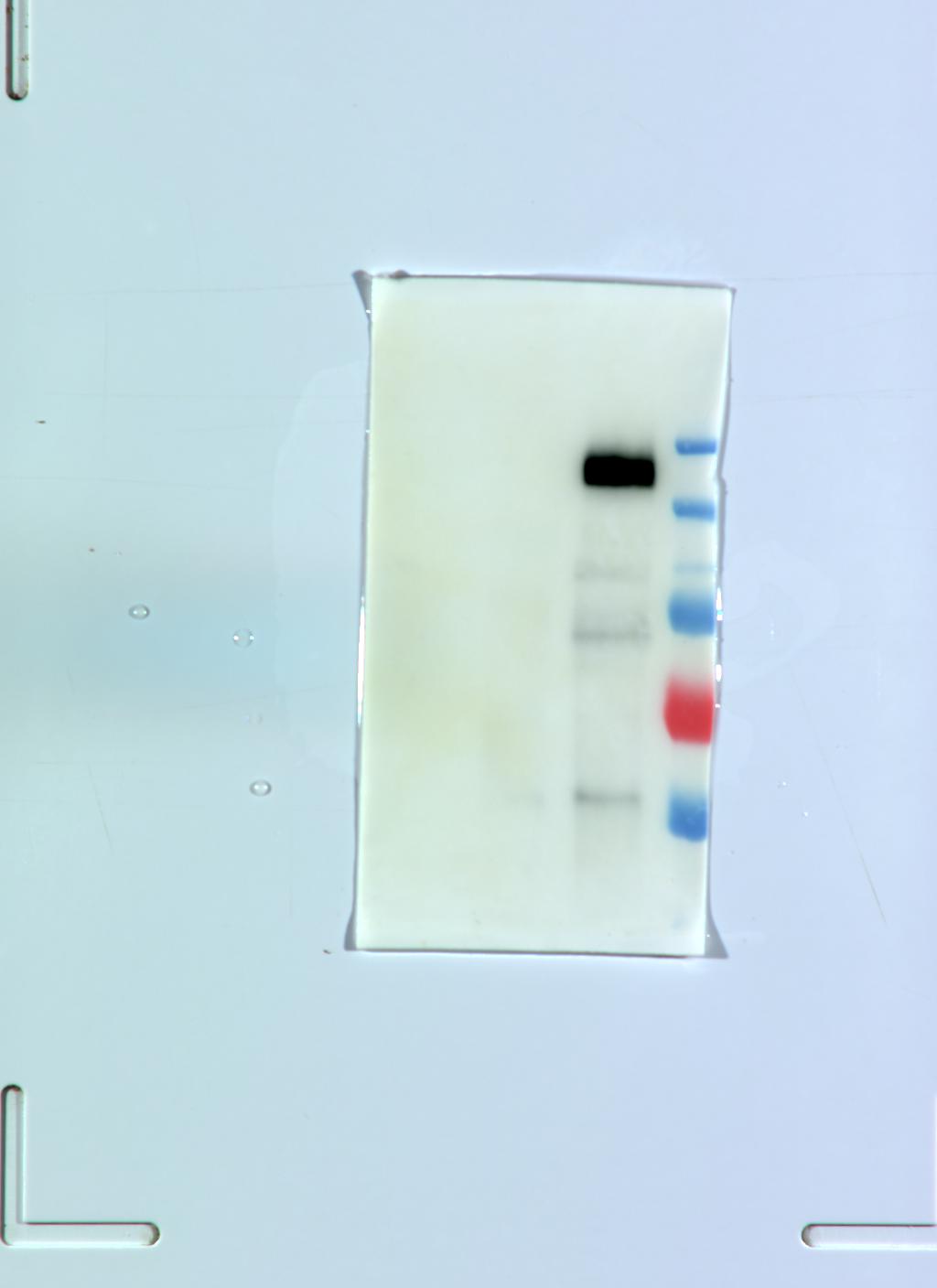


INPUT-anti-FLAG


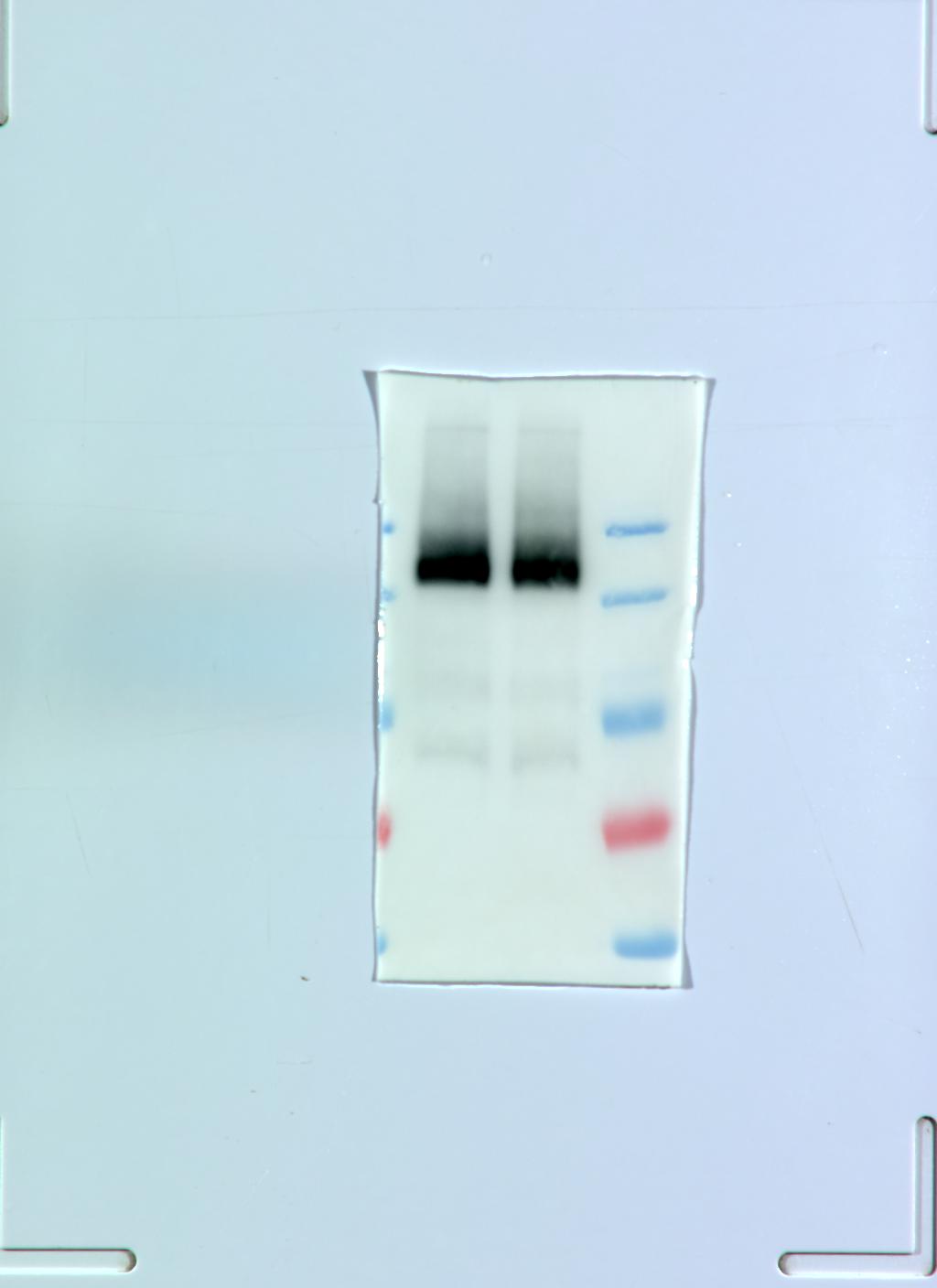


**FIGURES1:**

HU-ZBTB21-ANTI-HA


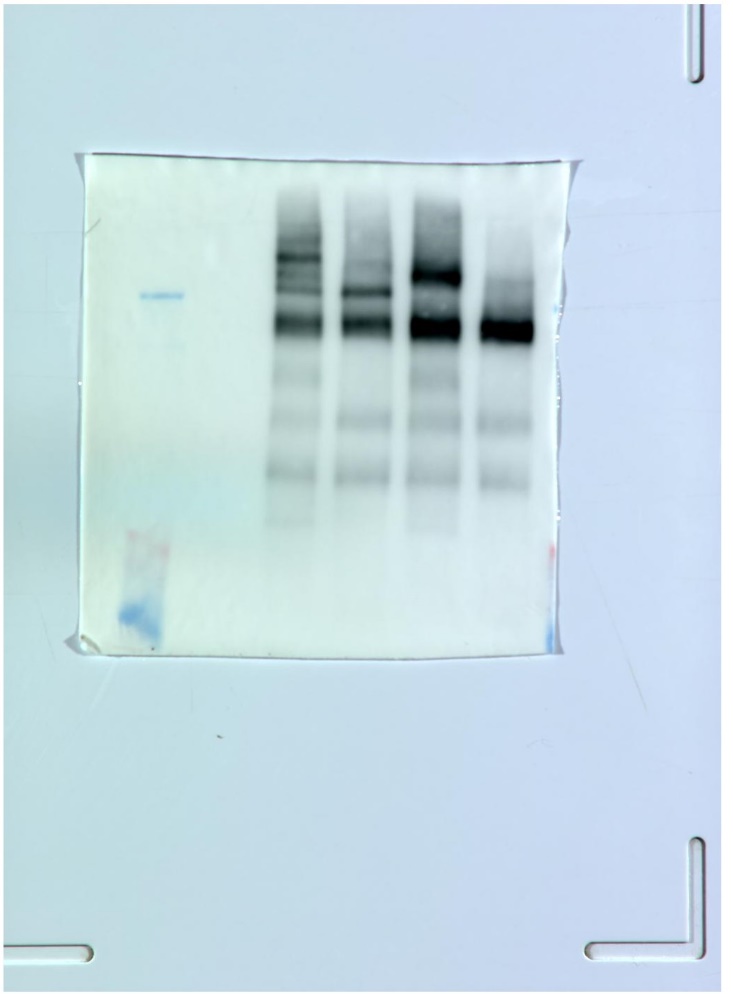


**FIGURES2:**

ANTI-HU-ZBTB21


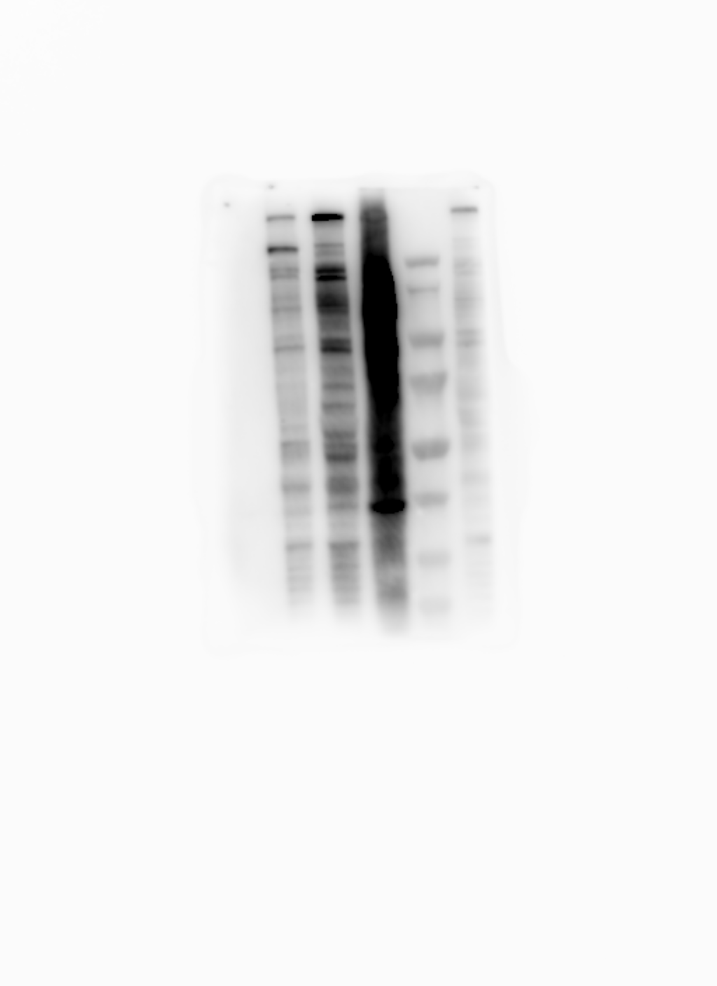

Supplement: Supplemental Information 1 [file peerj-12-17234-s001.docx]
