# Supplementary material for: SUMOylation of zebrafish transcription factor Zbtb21 affects its transcription activity
Source: PeerJ. 2024 Apr 22;12:e17234. doi: 10.7717/peerj.17234 (PMC11044885; doi:10.7717/peerj.17234)

**Figure4A：**

**IP:** **HA-Zbtb21-Zbtb14**

IP-Anti-HA（The following is a full-length blots）


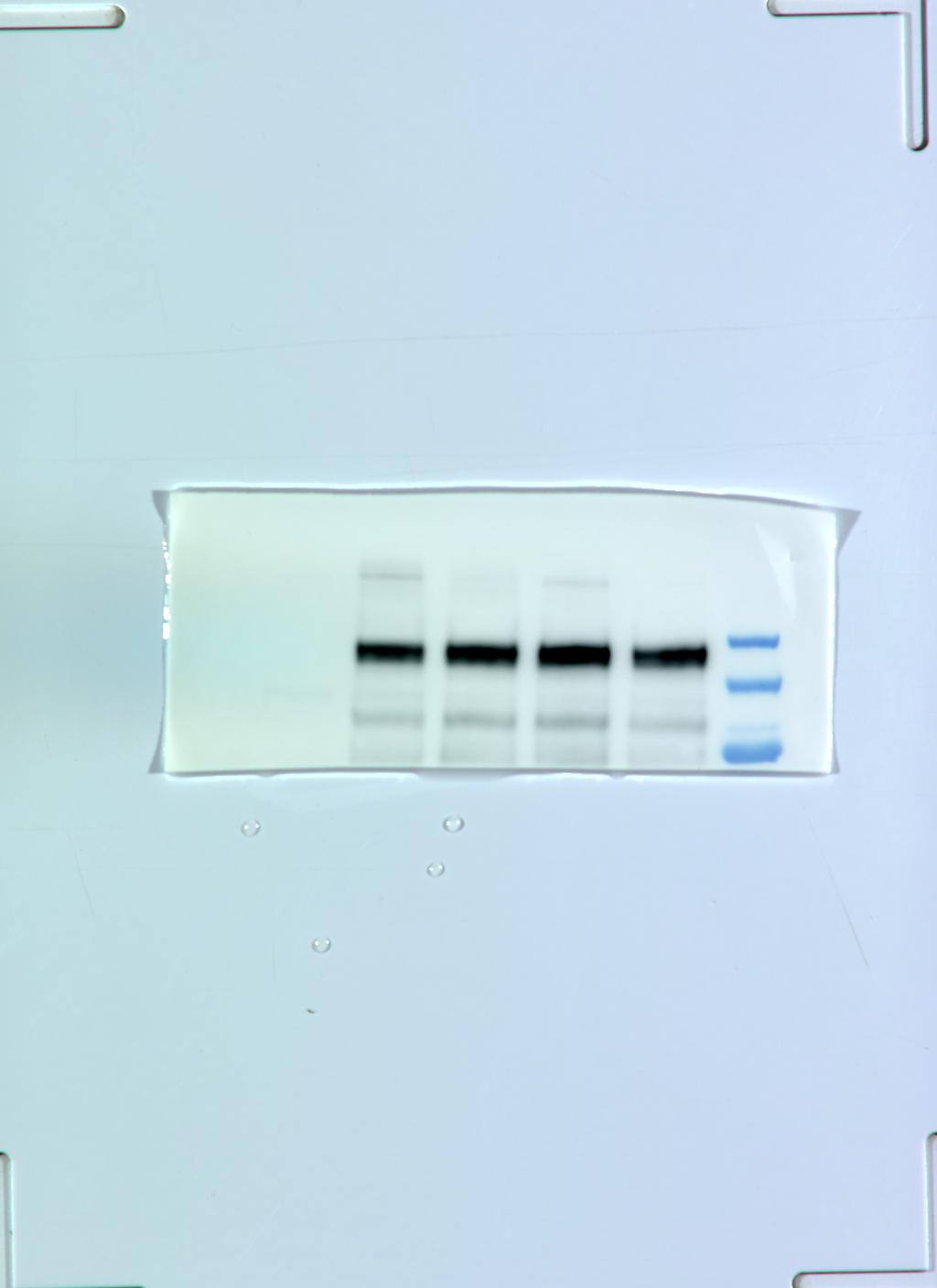


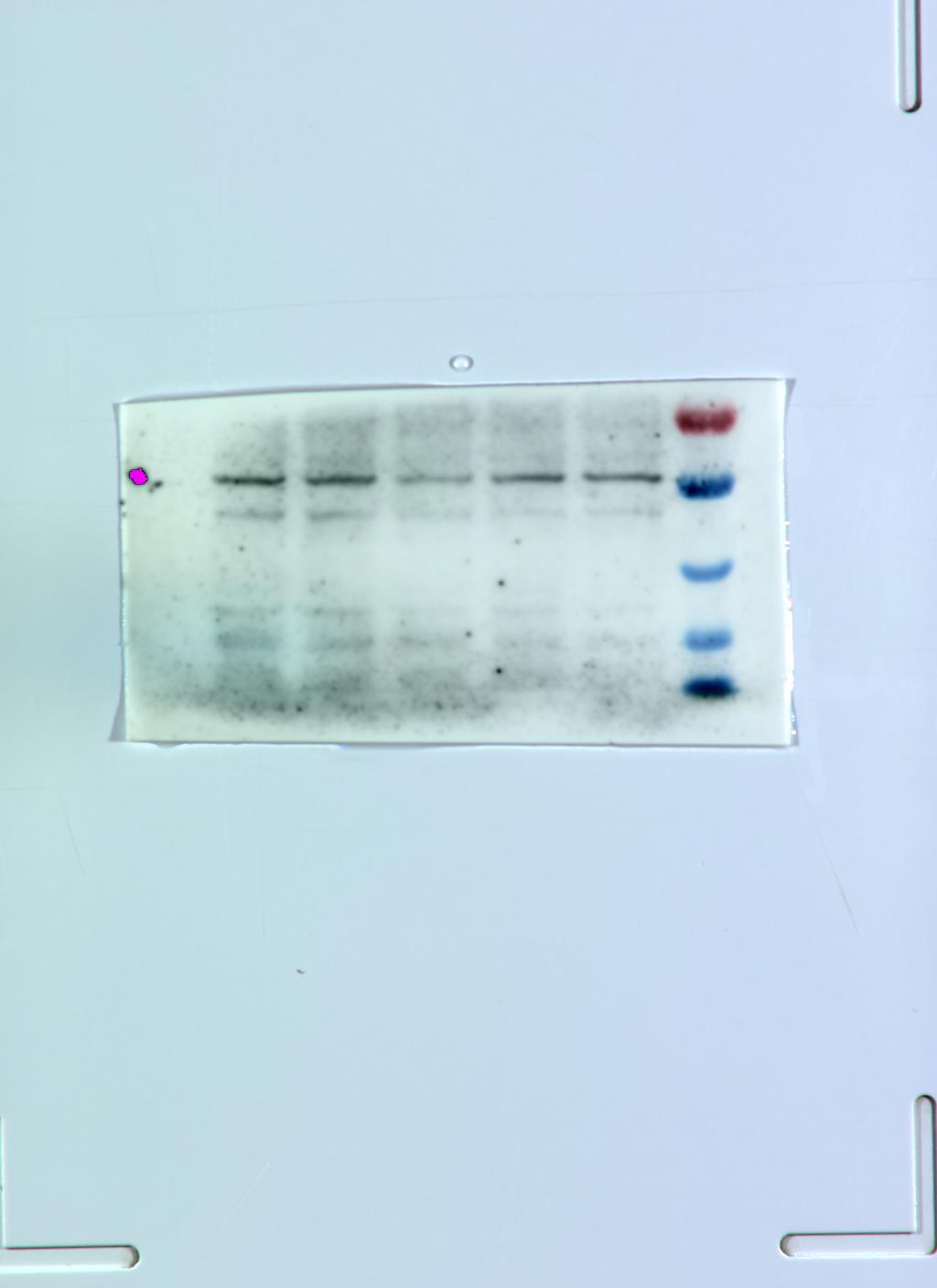


INPUT（The following is a full-length blots）

anti-HA


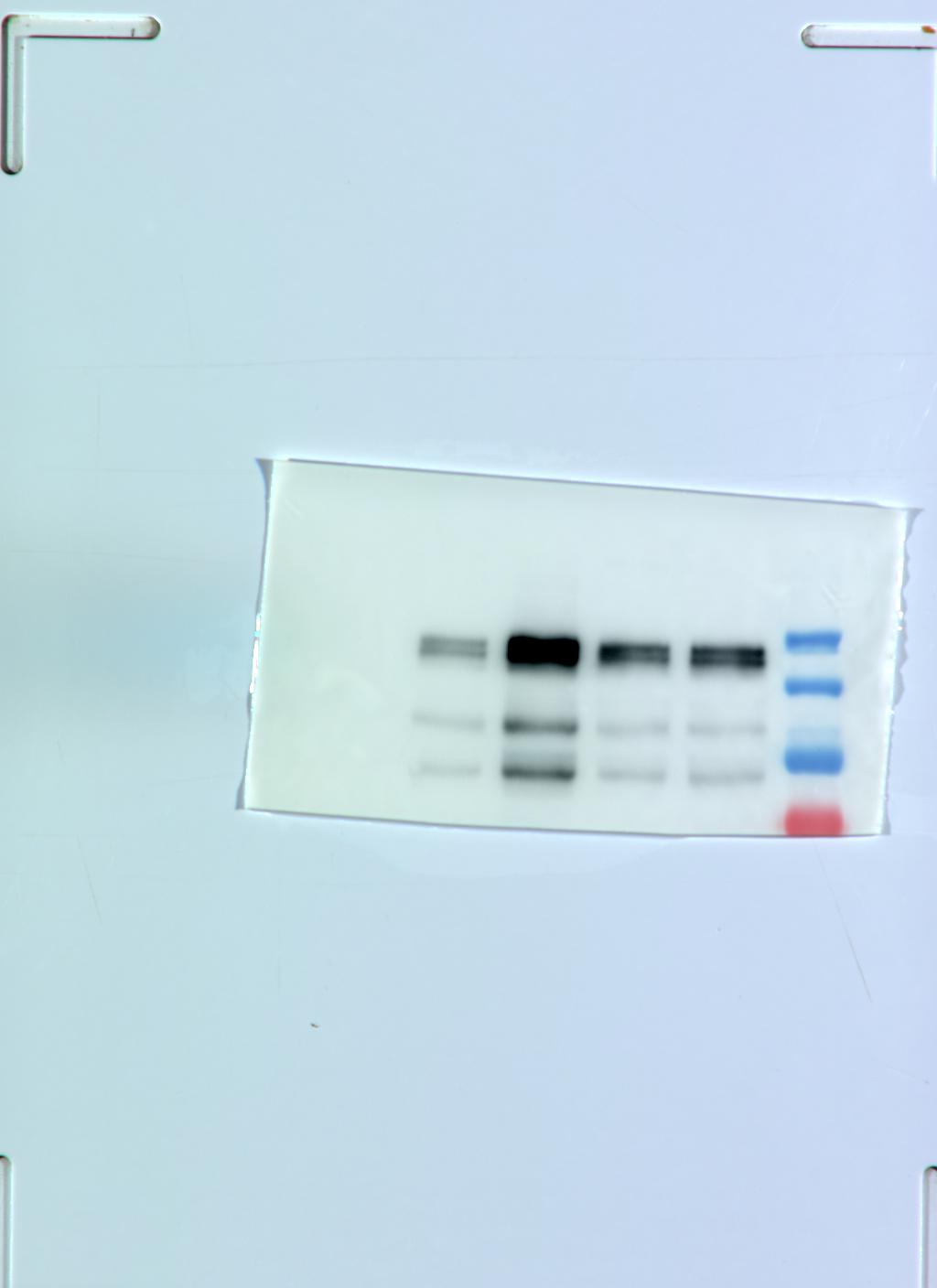


anti-flag


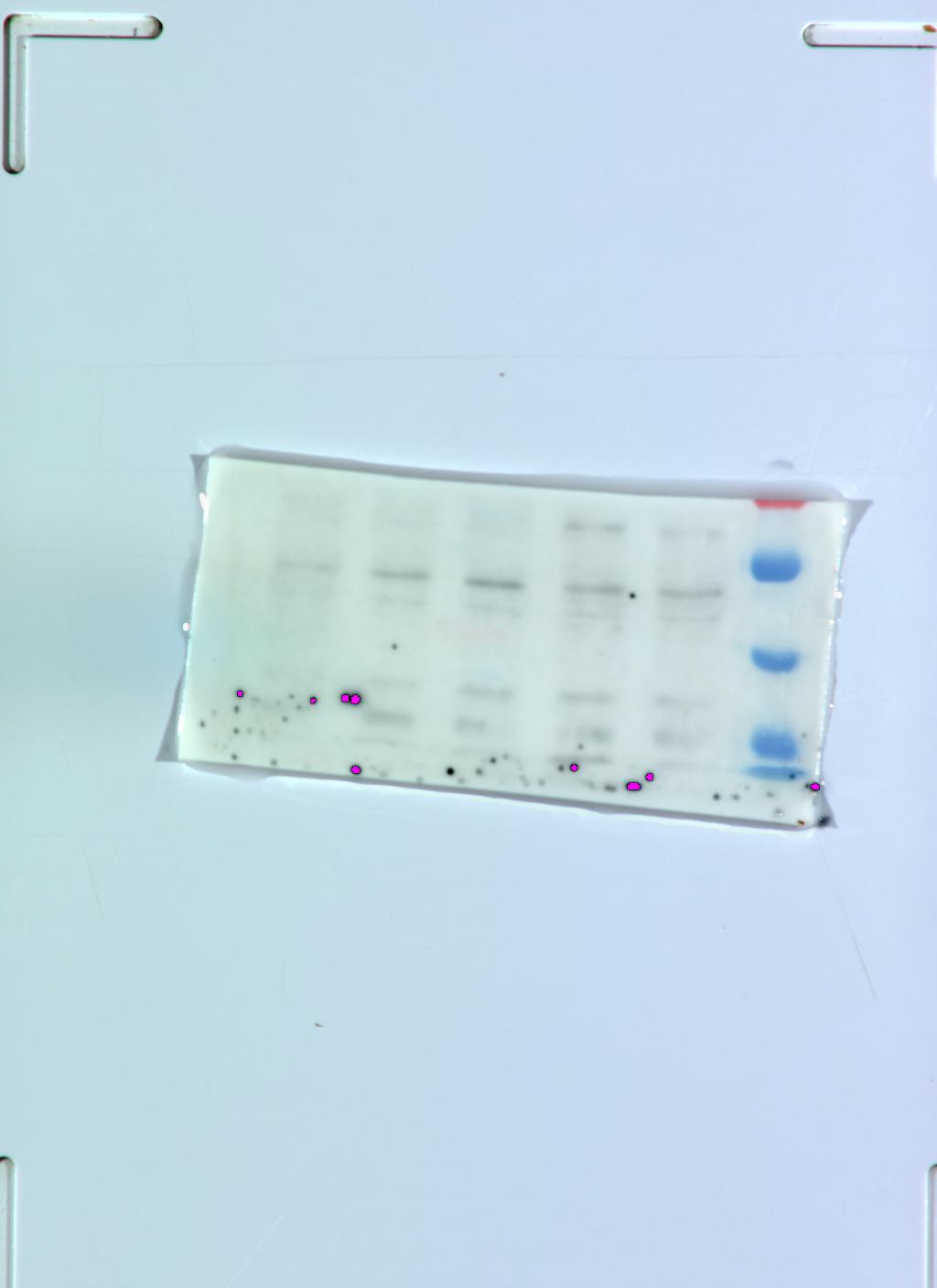

Supplement: Supplemental Information 3 [file peerj-12-17234-s003.docx]
