# Supplementary figures and images for: SUMOylation of zebrafish transcription factor Zbtb21 affects its transcription activity
Source: PeerJ. 2024 Apr 22;12:e17234. doi: 10.7717/peerj.17234 (PMC11044885; doi:10.7717/peerj.17234)

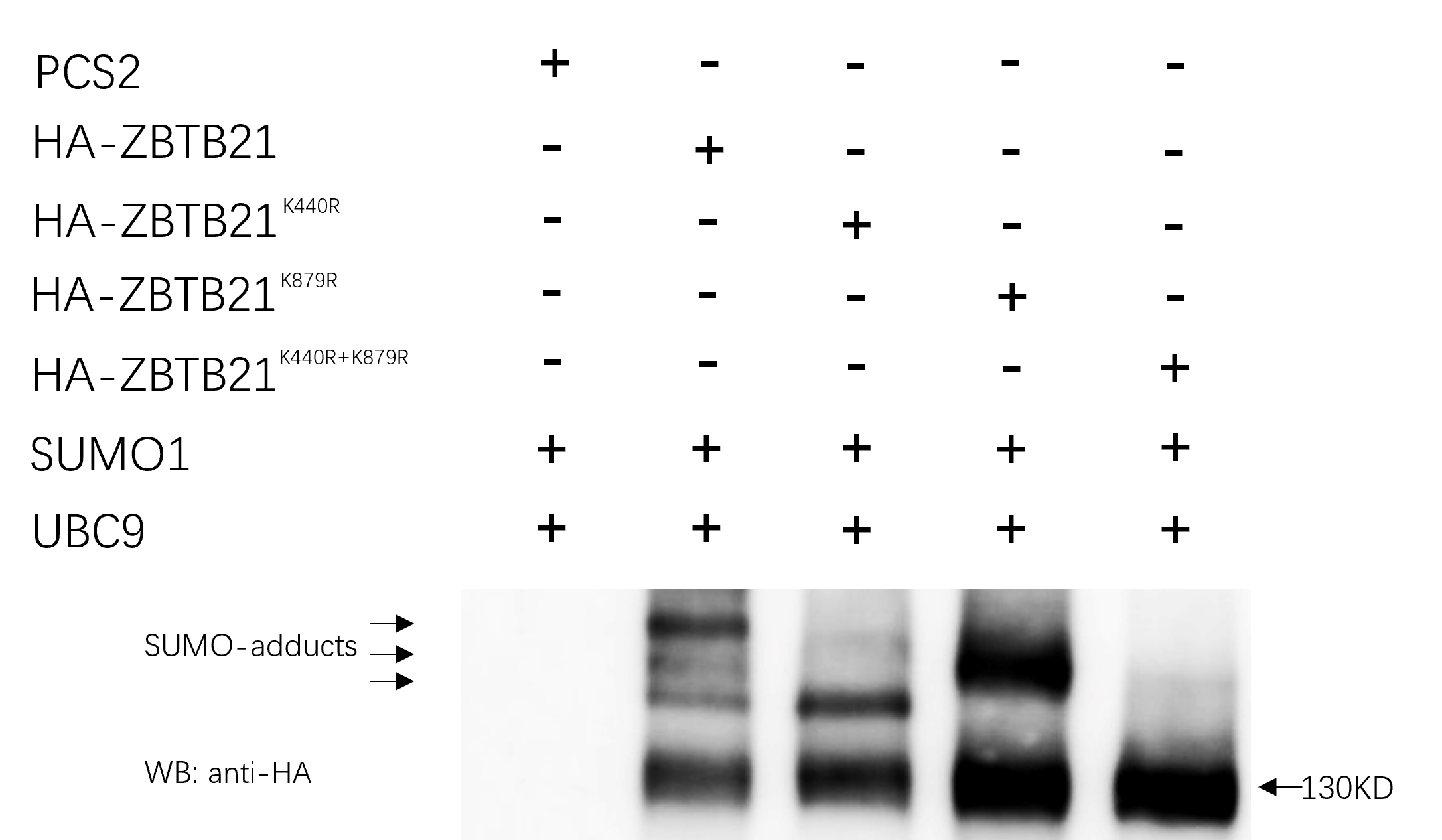

Supplement: Supplemental Information 4 — Western blot analyses (anti-HA) of HA-tagged wild type (WT), Z BTB 21K440R, ZBTB21K879R, and ZBTB21K440/879R mutant proteins in the presence of UBC9 and SUMO1 in HEK293T cells. [file peerj-12-17234-s004.jpg]

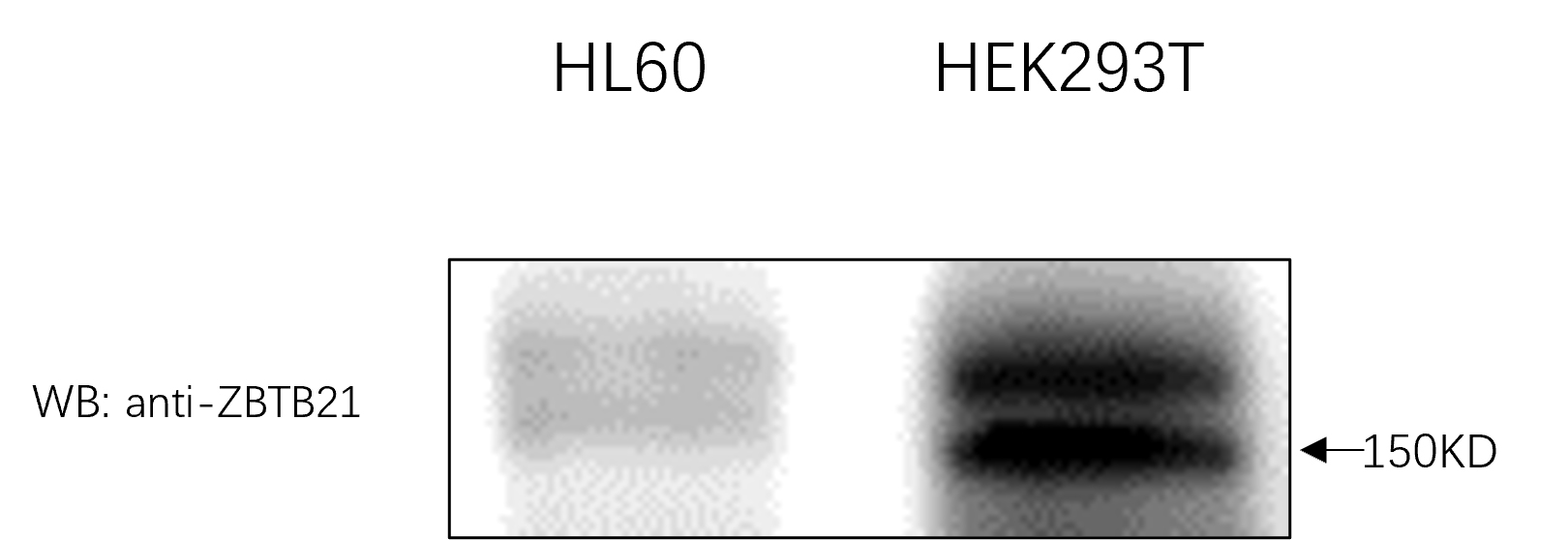

Supplement: Supplemental Information 5 — Western blot analyses to detect endogenous ZBTB21 in HL60 and HEK293T cell lines. [file peerj-12-17234-s005.jpg]
